# Prolonged Glucosuria With Sodium-Glucose Cotransporter-2 (SGLT2) Inhibitors: A Case Report and Review of Literature

**DOI:** 10.7759/cureus.11554

**Published:** 2020-11-18

**Authors:** Ankita Aggarwal, Anubhav Jain, Sonali Sachdeva, Zain I Kulairi

**Affiliations:** 1 Department of Internal Medicine, Wayne State University School of Medicine, Rochester Hills, USA; 2 Department of Cardiology, Ascension Genesys Hospital, Rochester Hills, USA; 3 Department of Medicine, Lady Hardinge Medical College, New Delhi, IND

**Keywords:** sglt-2 inhibitors, ketoacidosis, glucosuria, diabetes

## Abstract

Sodium-glucose cotransporter-2 (SGLT2) inhibitors assert their role as an anti-diabetic medication by reversibly inhibiting sodium-glucose cotransporters in the renal proximal tubules and resulting in enhanced glucose excretion. Due to their reversible effect on the transporters in the proximal tubule, it is expected that all their metabolic effects, including glucose excretion, should also cease in two to three days, as per their half-life of 10-15 hours. However, it is increasingly being observed that the glycosuric effect of SGLT2 inhibitors persists beyond this duration and, in many cases, exceeds their other known metabolic effects, which resolve sooner. We present a case report of a 53-year-old diabetic male who developed SGLT2 inhibitor-related euglycemic diabetic ketoacidosis (EuDKA) two days after being discharged post a laparoscopic appendectomy procedure. The patient was treated as per the recommended protocols, after which ongoing metabolic acidosis abated, but the patient’s urinary glucose remained on the higher end. We present an up-to-date review of existing evidence on this rare but serious side effect of SGLT2 inhibitors.

## Introduction

Sodium-glucose cotransporter-2 (SGLT2) inhibitors are a new class of Food and Drug Administration (FDA)-approved oral hypoglycemic medications for patients with type 2 diabetes. These drugs act by inhibiting the SGLT2 receptors in the proximal tubules, which are responsible for 90% of the reabsorption of glucose from urine. As a result, they decrease blood glucose levels by increasing the excretion of glucose in urine. In addition to their lowering blood glucose levels, these drugs have considerable cardioprotective and renoprotective effects. Initial evidence came from the Empagliflozin Removal of Excess of Glucose Outcome (EMPA-REG OUTCOME) trial [1] and the Canagliflozin Cardiovascular Assessment Study (CANVAS) [2] that revealed improved cardiovascular outcomes with empagliflozin and canagliflozin, respectively. Since then, multiple trials, like the Dapagliflozin Effect on Cardiovascular Events-Thrombolysis in Myocardial Infarction (DECLARE-TIMI) and the Canagliflozin and Renal Events in Diabetes with Established Nephropathy Clinical Evaluation (CREDENCE), have shown the benefit of this class of drugs in patients with heart failure and chronic kidney disease [3,4]. As a result, these drugs are now recommended as adjunctive therapy for glucose reduction in patients with established atherosclerotic cardiovascular disease, established kidney disease, or heart failure. With these new updates in the guidelines, the use of these drugs is expected to increase. However, recently there have been concerns about the association of SGLT2 inhibitors with a potentially life-threatening condition called euglycemic diabetic ketoacidosis (EuDKA), requiring immediate hospitalization. Consequently, the FDA released a warning regarding this complication. The overall incidence of EuDKA with SGLT2 inhibitor use has been estimated at 0.1% [5]. A study by Barski et al. compared the incidence and clinical characteristics of EuDKA in patients with type 1 and type 2 diabetes mellitus (DM) [6]. The incidence and severity of EuDKA were found to be higher in patients with type 1 DM than type 2 DM. Although the occurrence of EuDKA with SGLT2 inhibitor use is a well-known phenomenon, there is little knowledge about the mechanism behind its protracted glucose excreting effect. We begin with a case report and wind up with a discussion of possible explanations/potential mechanisms related to this drug effect.

## Case presentation

The patient discussed in this case report is a 53-year-old male with a past medical history of hypertension, hyperlipidemia, and diabetes mellitus type 2, managed on metformin and canagliflozin. After undergoing a routine laparoscopic appendectomy, his postoperative course remained uneventful. He started tolerating oral feeds, having bowel movements and remained hemodynamically stable. Therefore, he was discharged two days after the surgery. However, a few hours later, on the day of discharge, he presented to the emergency department with sudden onset generalized abdominal pain, non-exertional shortness of breath, and fever.

On presentation, he was febrile (temperature: 100.7°F), tachycardia (heart rate: 114 bpm), tachypneic (respiratory rate: 30/min), and hypoxic with an oxygen saturation of 87% on room air. His physical examination was benign; lungs were clear to auscultation bilaterally and soft, non-tender abdomen. The surgical site looked clean, without any discharge, and was non-tender to touch.

The patient’s initial laboratory measurements were all within normal limits: blood glucose 126 mg/dL, sodium 134 mmol/L, potassium 4.5 mmol/L, chloride 105 mmol/L, bicarbonate 17 mEq/L, blood urea nitrogen (BUN) 11 mg/dL, and creatinine 0.9 mg/dL. The exception was a high anion gap of 20.8 mEq/L and a pH of 7.21. The patient was started on four liters nasal cannula, switched to non-rebreather as he remained hypoxic, and was subsequently admitted to the hospital for further management. A septic workup was initiated, and the patient was placed on empiric antibiotics. 

Further laboratory results revealed positive serum ketones (beta-hydroxybutyrate 2.69 mmol/L), whereas the urinalysis revealed glucosuria (urine glucose > 1500 mg/dL) and ketonuria and no proteins. A urine culture was negative for any infectious growth. Serum lactate was 13 mg/dL. A computerized axial tomography (CAT) scan of the patient’s chest showed bilateral lower lobe consolidations suggestive of probable pneumonia. A CAT scan of his abdomen showed probable postoperative ileus with no obstruction or abscess. On further questioning, the patient reported having resumed canagliflozin and his regular diet as instructed upon being discharged.

Due to a high anion gap and positive ketones in the blood with normal blood glucose levels, the patient was diagnosed with euglycemic diabetic ketoacidosis secondary to his SGLT2-inhibitor use. Thus, canagliflozin was stopped, and the patient was started on an insulin drip with 10% dextrose. Subsequently, he was transferred to the intensive care unit (ICU) for closer monitoring. The patient’s condition started improving after the treatment with insulin and dextrose. On day two, following admission, his symptoms improved, and subsequent laboratory results also looked better with a lower anion gap of 16.2 mEq/L. On day three following admission, he was switched from an insulin drip to subcutaneous insulin and sliding scale as his laboratory results approached normal, with an anion gap of 13.2 mEq/L and beta-hydroxybutyrate of 0.81 mmol/L. With symptom resolution, the patient was then returned to the floor. However, his urine glucose remained high (>1500 mg/dL).

Discharged the next day and advised to continue subcutaneous insulin for a week, the patient was asked to consult his endocrinologist for further management. He was also recommended against resuming canagliflozin.

## Discussion

Euglycemic diabetic ketoacidosis is a rare but life-threatening condition associated with the use of SGLT2 inhibitors like canagliflozin. It is defined as a clinical triad of increased anion gap metabolic acidosis, ketonemia or ketonuria, and blood glucose levels below 200 mg/dL [7]. Few mechanisms have been proposed to explain this complication. SGLT2 inhibitors decrease the reabsorption of glucose from proximal renal tubules, lowering blood glucose levels. As a result of reduced blood glucose levels, insulin secretion by the pancreas is decreased.

Furthermore, insulin acts to increase lipolysis, resulting in free fatty acid production and subsequent conversion to ketone bodies by the process of β‐oxidation, which occurs in the liver. Also, SGLT2 inhibitors increase glucagon secretion directly on alpha cells or indirectly due to decreased insulin levels. Glucagon secretion contributes further to the overproduction of ketone bodies. This entire cascade of events is exacerbated when the patient is undergoing a stressful situation. Thus, most euglycemic diabetic ketoacidosis cases occur in the presence of precipitants such as surgery, extensive exercise, myocardial infarction, stroke, severe infections, prolonged fasting, and other taxing stressful physical and medical conditions [8]. 

Our case is unique because the patient developed ketoacidosis even though canagliflozin was appropriately stopped preoperatively, as directed, and only resumed postoperatively at the time of discharge when he returned to a regular diet. Considering that the half-life of canagliflozin is 10.6 hours and 13.1 hours for the 100 mg and 300 mg doses, it can be presumed that the drug's effect will not last more than two to three days [9]. Thus, the presence of high urine glucose 72 hours after stopping the medication, in this case, is an intriguing and puzzling finding.

In our case, prolonged glucosuria was not accompanied by prolonged ketonemia, as reported previously [10]. In this case, protracted ketonemia has been attributed to the lack of insulin administration. Contrary to this, our patient was promptly diagnosed as EuDKA and given optimal insulin treatment until normalization of ketoacidosis. As seen in both cases, the prolonged glucosuria highlights the protracted effect of SGLT2 inhibitors far beyond their half-life. 

Protracted glucosuria even after discontinuation of the drug seems to be an effect related to the drug class since it has also been reported with other SGLT2 inhibitors like empagliflozin, dapagliflozin, and ipragliflozin [10,11].

Several mechanisms have been proposed in the literature to explain these findings, mostly relating to the drug's pharmacological properties. 

Canagliflozin does not exhibit time‐dependent pharmacokinetics and is accumulated in plasma up to 36% following multiple doses [12]. This could potentially explain the lengthened glucose-excretory effect of canagliflozin, as evidenced in our patient. Also, the drug itself is known to delay the reversibility of SGLT2 inhibition compared to its short half-life [13]. 

Given the high (80-90%) and pH-dependent protein binding of SGLT2 inhibitors, it is reasonable to assume that SGLT2 inhibitors remain bound to plasma proteins in the acidic environment created as a result of EuDKA and dissociate once the acidosis resolves with treatment [14]. Further, the use of exogenous insulin administered to treat ketoacidoses may have contributed to prolonging glucose excretion by compounding the glucosuric action of SGLT2 inhibitors [15].

In their study, Liu et al. observed that urinary glucose excretion does not correspond to plasma levels of SGLT2 inhibitors and discussed possible pharmacological mechanisms for the same [16]. They hypothesized that these drugs are not just filtered but also actively secreted into the proximal tubule leading to higher than expected concentrations being delivered to the SGLT2 transporter location. This, coupled with the slow off-rate of SGLT2 inhibitors, could lead to sustained levels of the drug in the proximal tubule and resultant glucosuria even in the presence of low plasma concentration. Other plausible reasons include SGLT2 inhibitors' effect on estimated glomerular filtration rate (eGFR) [17] and a potential downregulation of their receptors [18]. Lastly, polymorphisms associated with uridine diphosphate glycosyltransferase (UGT1A9) [9], the metabolizing enzyme, can also result in prolonged urinary glucose excretion as a result of increased drug availability.

The enhanced glucose eliminating effect that continues even after stopping the drug has grave clinical implications for someone who is currently not on an SGLT2 inhibitor. In this scenario, diagnosing ketoacidosis will be challenging for the physician because of existing euglycemia/hypoglycemia. Since the increased glycosuria can lead to osmotic diuresis, dehydration should be well maintained during this period. Patients should be carefully monitored and given adequate insulin and carbohydrate until glycosuria resolves. A massive caloric loss in the form of glucose and the resulting dehydration can precipitate another ketoacidosis event. It has been postulated that maintaining adequate hydration in a patient presenting with ketoacidosis can aggravate glucosuria and osmotic diuresis [13]. Adequate oral intake in the postoperative period must be ensured to prevent EuDKA. There should be no hurry to restart the drug, especially in patients undergoing gastrointestinal surgery where chances of postoperative ileus are high. Preoperative fasting, a requirement for surgical procedures, could have precipitated canagliflozin-associated diabetic ketoacidosis in our patient who underwent laparoscopic appendectomy. Thus, stopping the drug 24 hours before undergoing stressful situations such as surgery may not be enough. There is a need to maintain high diagnostic suspicion during the perioperative period in patients with a history of SGLT2 inhibitor intake, despite ceasing its use 24 hours before the surgery. There have been growing concerns for this perioperative dilemma, and some suggest stopping the drug earlier than 24 hours and taking time to restart it post-surgery. Given this, the FDA has revised safety labeling changes for SGLT2 inhibitors and recommended stopping using them three to four days before surgery [19]. A study also suggested that the decision to stop and restart the drug be approached in a more individualized and patient-appropriate manner, giving due consideration to both patient and surgical factors [20]. 

The absence of hyperglycemia makes the diagnosis challenging. Physicians need to look for ketones even with normal glucose levels in any patient taking an SGLT2 inhibitor and presenting with nausea, vomiting, abdominal pain, or shortness of breath, especially when precipitating factors like recent surgery and/or infection, as in our case. 

## Conclusions

SGLT2 inhibitor-induced urinary glucose excretion is a prolonged effect with delayed recovery, and in many cases, continues even after cessation of associated metabolic derangement. Although there are insufficient studies to ascertain the precise mechanism, it is a significant finding in patients suffering from SGLT2 inhibitor-induced euglycemic ketoacidosis. If not taken care of, continuous glucose excretion and resultant caloric loss can not only prolong recovery but also lead to recurrent ketosis and related complications. Large-scale studies are warranted to determine the actual mechanism involved to re-affirm the safety of this widely used class of drugs. 
